# Development and characterization of two cell lines from gills of Atlantic salmon

**DOI:** 10.1371/journal.pone.0191792

**Published:** 2018-02-14

**Authors:** Mona C. Gjessing, Maria Aamelfot, William N. Batts, Sylvie L. Benestad, Ole B. Dale, Even Thoen, Simon C. Weli, James R. Winton

**Affiliations:** 1 Norwegian Veterinary Institute, Oslo, Norway; 2 US Geological Survey Western Fisheries Research Center, Seattle, Washington, United States of America; 3 Norwegian University of Life Sciences, Oslo, Norway; INRA, FRANCE

## Abstract

Gill disease in Atlantic salmon, *Salmo salar* L., causes big losses in the salmon farming industry. Until now, tools to cultivate microorganisms causing gill disease and models to study the gill responses have been lacking. Here we describe the establishment and characterization of two cell lines from the gills of Atlantic salmon. Atlantic salmon gill cell ASG-10 consisted of cells staining for cytokeratin and e-cadherin and with desmosomes as seen by transmission electron microscopy suggesting the cells to be of epithelial origin. These structures were not seen in ASG-13. The cell lines have been maintained for almost 30 passages and both cell lines are fully susceptible to infection by infectious hematopoietic necrosis virus (IHNV), viral hemorrhagic septicemia virus (VHSV), infectious pancreatic necrosis virus (IPNV), Atlantic salmon reovirus TS (TSRV) and Pacific salmon paramyxovirus (PSPV). While infectious salmon anemia virus (ISAV) did not cause visible CPE, immunofluorescent staining revealed a sub-fraction of cells in both the ASG-10 and ASG-13 lines may be permissive to infection. ASG-10 is able to proliferate and migrate to close scratches in the monolayer within seven days *in vitro* contrary to ASG-13, which does not appear to do have the same proliferative and migratory ability. These cell lines will be useful in studies of gill diseases in Atlantic salmon and may represent an important contribution for alternatives to experimental animals and studies of epithelial–mesenchymal cell biology.

## Introduction

Gill diseases are among the most serious economical and welfare threats to the farming of Atlantic salmon, *Salmo salar* L. More knowledge of gill diseases is urgently needed, but a major obstacle is that most agents associated with gill diseases have not been cultured. These include *Candidatus* Branchiomonas cysticola [1], *Candidatus* Piscichlamydia salmonis [2], *Desmozoon lepeophtherii* [3] and salmon gill poxvirus [4], all of which appear to be epitheliotropic. Controlled experiments to assess the role each play in relation to gill diseases have thus not been performed. Current studies are descriptive [5–8], and difficult to interpret.

The mucosal surfaces, with the epithelial layer form a barrier between the environment and the internal organs. For the gills, the epithelium must provide both protection and have the ability to exchange ions. Hence, desmosomes and adherent junctions provide the strong bonds necessary to maintain cellular tight junctions which regulate paracellular permeability [9]. Mucosal damage, as a result of infectious agents or mechanical injury, results in an epithelial response. Closing of the damaged monolayer involves proliferation and migration of epithelial cells [10], features that are difficult to study *in vivo*. A main host response in gill diseases in Atlantic salmon is an exaggerated proliferation of the epithelial cell layer. This proliferation may form a defensive barrier. However, it may also cause severe respiratory problems, by increasing the distance between the blood and the water, thus impairing the efficient transport of oxygen [11]. Surprisingly, very few studies have been reported on the epithelial proliferation that may have a profound impact on the health of the fish, regardless of the exact, inciting cause. Establishment of gill cell lines from Atlantic salmon can contribute as a cultivation system for the many pathogenic epitheliotropic gill microorganisms and as a model system to investigate specific cell responses in the gills [12]. Here, we report the development and characterization of two novel cell lines from Atlantic salmon gill and their potential to propagate different fish viruses.

## Materials and methods

### Animal husbandry and ethical considerations

Gills for the development of primary cells were obtained from juvenile Atlantic salmon weighing about 50 g. The handling of the live fish was done in accordance with Norwegian regulation of experimental animal procedures and thus fully anesthetized using Benzoak (ACD Pharmaceuticals, Oslo). The fish were part of another experiment, approved of by the local Institutional Animal Care and Use Committee (IACUC). FOTS ID: 6406 and we used the control fish of that experiment. The IACUC approved that additional sampling from the fish for our purpose could be performed after the fish were euthanized in the original experiment–*i*.*e*. the fish did not experience any additional stress inflicted by the sampling.

Fish were reared in fresh water at 12°C. Photoperiod was maintained at a constant 14 h light/10 h dark cycle and fish were fed a daily 1% (w/w) ration of fish chow. Prior to euthanasia the fish were kept in fresh water with 20 ppm Chloramine for 30 minutes. Fish sampling for preparation of primary gill epithelial cells were performed according to internationally recognized ethical guidelines. The use of cells, including the use of primary cells, in research is preferred by NARA as this is in compliance with the guiding principles for more ethical use of animals in research: the Three Rs- replacement, reduction and refinement, in particular the reduction in the number of animals used in research.

### Development of primary cells

Subsequent work was performed with room tempered liquids. The gills were excised and transferred to a 50-ml tube and incubated on a rotator with fresh Hanks’ balanced salt solution (HBSS, Lonza, Belgium) for 10 minutes, five times. The work was thereafter carried out in a laminar flow hood. Sterile techniques were used throughout all cell culture procedures. The gill cartilage was removed. Then the filaments were cut by a scalpel in explants of 1–2 mm on a sterile petri dish supplemented with a few droplets of HBSS. From this pool of explants, 4–6 explants were plated in each well of a 6-well CellBIND plate (Corning, US) with a small amount of medium to allow the tissue pieces to adhere. The medium was denoted L15^3+^ and consisted of Leibovitz L-15 (Lonza) supplemented with non-essential amino acids (1% 100x, Lonza), Na-pyruvate (1% 100x, Lonza), insulin, transferrin and selenium (ITS, 0.2% 500x, Lonza), 4 mM L-glutamine (Lonza), 0.05 mg/ml gentamycin (Lonza), 0.03 mM 2-mercaptoethanol (Gibco, US) and 20% fetal bovine serum (FBS superior, Biochrom, Germany). The plate was incubated at 15°C. After one day, when the explants had adhered to the surface of the well, additional L15^3+^ was added to the chambers. In some wells, cells migrated out from the gill explants. In one well, prominent growth of cells was seen and most vigorous from the edge cut by the scalpel. In this well, a continuous outgrowth of cells with varying morphology was seen as early as one day. Subsequent work was carried out on this culture. The culture continued to grow and after two weeks, a confluent monolayer had developed. The culture was then washed three times with PBS (Lonza), and incubated with 200 μl of trypsin (Lonza) for approximately 3 minutes to detach the cells. The plate was gently agitated and the trypsinization process was observed visually using a phase contrast microscope. The cells were resuspended in 4 ml of L15^3+^, then divided between 2 wells in a 6-well CellBIND plate (Corning). The cells continued to divide, and after another 10 days a confluent monolayer had developed in the two wells. The two wells were washed with PBS and incubated with trypsin as previously described and the cells in each well were resuspended in 3 ml of L15 ^3+^.

### Development of two cell lines

The two different suspensions of cells were transferred to 25 cm^2^ CellBIND flasks (Corning) and incubated at 15°C. During the next days, only a few aggregations of cells developed and the growth slowed down. In the course of the next months, the cell aggregations fused, and gradually a confluent monolayer of fibroblast-like cells developed. The cells were washed three times with PBS and incubated with 1 ml of trypsin while inspected under the microscope. After the cells detached, 11 ml of L15^3+^ was added and the cells were divided between to two new 25 cm^2^ flasks (a total of 6 ml per flask). In the original flask cells with fibroblast-like morphology remained attached to the flask after trypsinization and 6 ml of L15^3+^ was added to this flask. Subsequent sub-culturing was performed with medium denoted L15^+^ consisting of Leibovitz L-15 supplemented with, 4 mM L-glutamine, 0.05 mg/ml gentamycin, 0.03 mM 2-mercaptoethanol and 10% FBS and resulted in the continuous propagation of two cell lines, each with different morphology. After passage 4, the cells were transferred to and cultivated in Nunc^™^ cell culture-treated EasYFlasks^™^ (Thermo Scientific, US) and kept at 20°C.

### Routine maintenance, harvesting and cryopreservation

When a confluent monolayer had developed, the cells were washed three times with PBS and incubated with 1 ml of trypsin. The flasks were gently agitated and the trypsinization process was observed visually using a phase contrast microscope. After addition of 11 ml of L15^+^, the cell suspension was divided between two new flasks. Culture flasks at passage 13, (Atlantic salmon gill cells 10, ASG-10) and 9 (ASG-13) with cells in vigorous growth, were frozen and then thawed and revitalized as described earlier [13].

### Documentation of cell morphology

Revitalized cells were inspected daily and split regularly as previously described. Staining of cells with Giemsa was performed as previously described [13] on confluent cells in passage 15 and 19 (ASG-10) and 11 and 20 (ASG-13). Briefly, cell medium was removed from the flasks and cells were washed in PBS, fixed in methanol, stained with Giemsa for 2 min, washed with water and inspected in a phase contrast microscope.

#### Immunostaining

For further characterization, the cells were grown on sterile coverslips in 24-well plates. After 2 weeks, they were stained with antibodies to two different adherent junction proteins, *i*.*e*. e-cadherin, monoclonal IgG2α (BD Transduction Laboratories^™^, US) and ZO-1, polyclonal rabbit IgG (LSBio, Inc, US) and also to pan-cytokeratin, monoclonal IgG1 (Abcam, UK) to investigate their epithelial origin. Briefly, the cell medium was removed, then the cells were fixed in ice-cold acetone for 10 minutes, air dried and washed 3 times in PBS. Blocking was achieved with 5% skimmed milk in PBS for 30 min. The cells were then incubated with primary antibodies for 60 min. Alexa Fluor 488 conjugate (Invitrogen, US) were used as secondary antibodies, and cells incubated for 60 min. To visualize nuclei, the cells were incubated in propidium iodide for 5 min. The whole staining procedure was performed in the 24-well plates at room temperature. After staining, the cover slips were removed and mounted with SlowFade^®^ (Invitrogen) on slides, and imaging was performed on a Zeiss LSM 710 confocal microscope, using 488 nm and 561 nm lasers.

#### Transmission electron microscopy

For transmission electron microscopy (TEM) the cells were grown on hanging Millipore cell culture inserts (Merck, Germany) for four weeks and treated as previously described [4].

### Species identification by DNA barcoding

Samples of the new cell lines ASG-10 and ASG-13 were softly pelleted at 250 x g for 1 min, then most of the fluid removed and a 1 ml volume of TriZol (Ambion, Inc, US) added to each tube and mixed by rocking for 5 min. This lysate was transferred to a new 1.5 ml tube containing 100 μl of 1-bromo-3-chloropropane, mixed by inversion for 15 sec and then allowed to settle for 10 min at room temperature. A 12,000 x g centrifugation for 15 min at 4°C allowed separation of the aqueous RNA phase from DNA/protein. In order to obtain DNA from the interphase/organic phase, a 0.3 ml volume of 100% ethanol was added, mixed by inversion, incubated 2–3 min, then DNA pelleted at 2000 x g for 5 min at 4°C. Supernatant was removed and the DNA pellet washed three times with 1 ml of 0.1 M trisodium citrate containing 10% ethanol, with the DNA incubated at least 30 min between each wash and 2000 x g spin. After the wash steps, the DNA pellet was resuspended in 1.5 ml of 80% ethanol, incubated 10 min at room temperature, then pelleted again. The DNA pellets were dried for 5 min in a vacuum drier, then resuspended by pipetting with 0.5 ml of 8 mM NaOH, pelleted at 12,000 x g for 10 min and 450 μl DNA solution transferred to a new tube containing 86 μl of 0.1 M HEPES. A 5 μl DNA sample was prepared in a 50 μl total reaction mix, similar to that described by Winton *et al*. (2010) [14] including primers designed to amplify the cytochrome oxidase subunit I (COx1) except the GoTaq polymerase reagents (Promega, Inc.) were used at annealing temperatures of 50°C or 40°C. Although the Atlantic salmon COx1 sequence obtained from Genbank differed by 1–2 nt at the fathead minnow primer binding sites, the primers described in Winton *et al*. (2010) [14] were used for the ASG-10 and ASG-13 cellular DNA. Purified PCR products obtained were analyzed by Applied Biosystems Big Dye terminator chemistry on a 3130 Genetic Analyzer and nucleotide sequences compared.

### Virus susceptibility

Two 96-well plates were seeded with Chinook salmon embryo cells (CHSE-214), Epithelioma papulosum cyprini cells (EPC), Atlantic salmon kidney (ASK), ASG-10 and ASG-13 in L-15 medium. The EPC cells were incubated at 20°C and the other cells at 15°C. 100 μl of cell suspension was added to each well and incubated overnight. Seven, 10-fold serial dilutions of stock cultures of infectious hematopoietic necrosis virus (IHNV—WRAC strain), viral hemorrhagic septicemia virus (VHSV—F1 strain), infectious pancreatic necrosis virus (IPNV- SP strain), Atlantic salmon reovirus TS (TSRV—Tasmanian strain), Pacific salmon paramyxovirus (PSPV—Trask strain) and infectious salmon anemia virus (ISAV—Bremnes strain) were made and 50 μl of each virus dilution was added to each of 4 wells of seven rows of a 96-well plate of each cell line and incubated at 15°C. Sterile L-15 was used as negative control. Wells were scored at 7, 14 and 21 days and wells with typical cytopathic effect (CPE) were marked. TCID_50_ was calculated as previously described [15] and expressed as log_10_ TCID_50_ per 50 μl.

#### Immunostaining for ISAV

To investigate the apparent lack of permissiveness to ISAV further, two 24-well plates with coverslips were plated with ASG-10 (passage 16) and ASG-13 (passage 18). ASK II (Atlantic salmon kidney) cells were included as a control. One ml of cell suspension was added to each well and incubated at 20°C overnight. Three isolates of ISAV were chosen based on their ability to cause varying degree of disease in Atlantic salmon. Glesvær/2/90 is highly virulent, Can/F679/99 medium virulent and FO/121/14 low virulent [16, 17]. Four wells of each cell type were inoculated with 100 μl of each virus suspension. Four wells of each cell type were mock infected with sterile cell culture media. After 2 hours the virus suspension and cell culture media was removed and the cells were washed gently with PBS before fresh L-15^+^ media with 2% FBS was added. The cells were incubated at 15°C. CPE was examined after 2, 4 and 6 days post-infection in the ASK II cells. Six days post infection CPE was becoming noticeable in the control ASK II cells. Cell culture media from the inoculated ASG-10 and ASG-13 cells was passed to ASK II cells prior to fixation to confirm that viable virus particles were produced. Immunostaining for ISAV on ASG-10 and ASG-13 was performed as described above using a monoclonal antibody to ISAV nucleoprotein (NP- Sterling, UK) and alkaline phosphatase and permanent red for visualization (EnVision, Agilent, USA).

#### qPCR for ISAV

To further investigate ISAV permissiveness ASG-10 and ASG-13 were grown in 25 cm^2^ Nunc^™^ cell culture-treated EasYFlasks^™^. When the cells were confluent they were split 1:1. After 24 hours at 20°C the cells were rinsed twice with PBS and Glesvær/2/90, Can/F678/99 and FO/121/14 were allowed to adsorb to the cells at 15°C. Uninfected negative controls were adsorbed with L-15 media. After 2 hours a total of 8 ml of L15^2%^ consisting of L-15 supplemented with 4 mM L-glutamine, 0.05 mg/ml gentamycin, 0.03 mM 2-mercaptoethanol and 2% FBS. After gentle agitation 250μl of media was removed and kept at -80°C. After 3 and 7 days at 15°C 250μl of media was removed from each flask and frozen at -80°C. Lysis buffer was added to all samples and total RNA was extracted using NucliSenc^®^ easyMAG^™^ according to manufacturer protocol. The concentration of RNA was measured using NanoDrop ND-1000 (Saveen). RT-qPCR was performed for ISAV segment 8 as previously described [18].

### Cell culture migratory and proliferative abilities

Migratory and proliferative abilities were investigated on confluent cultures of ASG-10 (passage 18) and ASG-13 (passage 19) as previously described [19], with minor modifications. A sterile 10 ml pipette tip was pressed firmly against the bottom of the culture flask. Swiftly a vertical scratch was made down through the cell monolayer. Three separate scratches were made in each flask using varying degree of pressure, to ensure that a scratch was made but the surface of the flask was not damaged. Media and cell debris were carefully aspirated, and fresh media was added carefully against the wall of the flask. The cells were kept at 20°C, and were inspected daily for 7 days for the scratch to close using an inverted microscope. Pictures were taken at regular intervals. The distance from one side of the scratch to the other was measured in cm in the pictures, and the cell behavior was noted.

## Results

### Primary cell lines developed

We developed two adherent cell lines with different, predominant morphologies, ASG-10 and ASG-13. Upon trypsinization, the cells detached after 2–3 min. The cells in ASG-10 detached as single cells, while the cells in ASG-13 detached in groups and appeared elastic. ASG-10 and ASG-13 have now been passaged 29 and 28 times, respectively. When revitalizing the cells after cryopreservation, a good yield was achieved of both cell lines, and the cells became confluent after approximately 3 days.

#### Cell morphology

**Fig 1:**
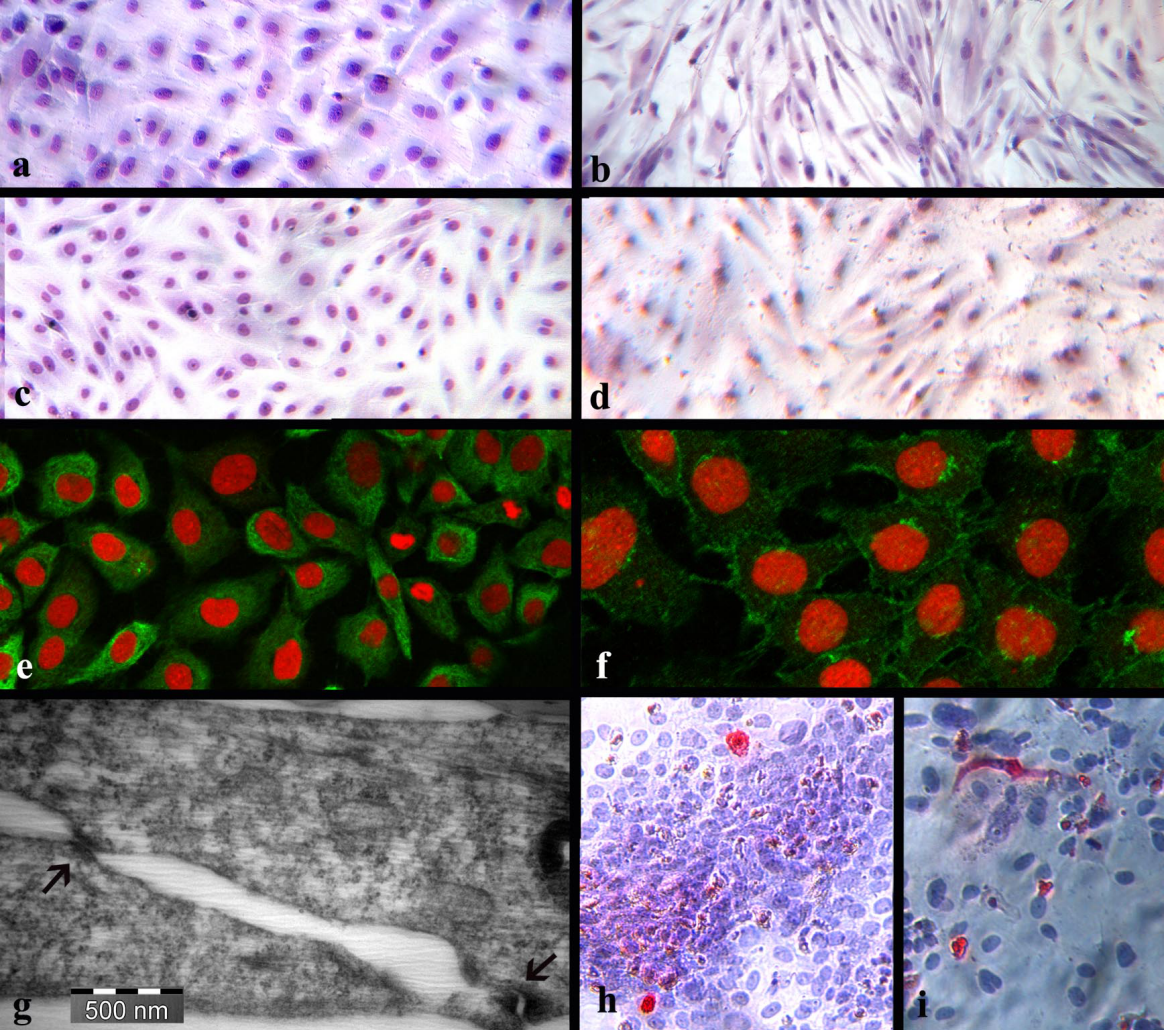
Staining and TEM of the cell lines. Cell lines from Atlantic salmon gills—ASG-10 (a, c, e-h) and ASG-13 (b, d and i). a and c: ASG-10 in passage 15 and 19 demonstrating polygonal cells (Giemsa). b and d: ASG-13 in passage 11 and 20 with elongated cells (Giemsa). e: Cytoplasmic staining showing cytokeratin in ASG-10. f: Staining of cell membranes showing e-cadherin in ASG-10. g: TEM showing the presence of desmosomes (arrow) in ASG-10. h-i: nuclear and cytoplasmic staining of some cells using antibody directed to ISAV nucleoprotein in ASG-10 (h) and ASG-13 (i).

ASG-10 consists of flattened, polygonal cells, with regular dimensions (Fig 1a and 1c) suggesting an epithelial nature, while ASG-13 cells have a bipolar or multipolar elongated spindle shape (Fig 1b and 1d) suggesting a fibroblastic nature. For several days after revitalization, prior to confluency, both cell lines appeared elongated.

Confluent cultures of ASG-10 consisted of cells with cytoplasmic staining for cytokeratin (Fig 1e), and cell membrane staining for e-cadherin (Fig 1f) and ZO-1. Desmosomes were demonstrated by TEM (Fig 1g) confirming their epithelial origin. Confluent cultures of ASG-13 consisted of a few cells staining for cytokeratin, but not for e-cadherin or ZO-1. Desmosomes were not detected in ASG-13.

### Species identification by DNA barcoding

The cell lines ASG-10 and ASG-13 were confirmed to be from Atlantic salmon by DNA sequencing of the COx-1 gene. No amplification was obtained when thermocycler was set at 50°C annealing temperature, probably due to slight primer mismatches. However, PCR was successful when the PCR primer annealing temperature was reduced to 40°C and bands of the expected 620 bp size were visible on the agarose gel. The 532 nt of trimmed sequences of both cell lines were identical to each other and were 100% identical to *Salmo salar* Genbank database submission JX960941.

### Virus susceptibility

Virus susceptibility of the new cell lines was compared with established lines based on the calculated titers of stock cultures of IHNV, VHSV, IPNV, TSRV, PSPV and ISAV in CHSE-214, EPC, ASG-10, ASG-13 and ASK cells. An infected well was scored as positive when typical CPE was observed and the titer expressed as log_10_ TCID_50_ per 50 μl. The ASG-10, ASG-13 and CHSE-214 cell lines were susceptible to IHNV, VHSV, IPNV, TSRV, and PSPV, while the EPC line was permissive for IHNV, VHSV and TSRV (Table 1). Although the ASK cell line showed good sensitivity to ISAV, no CPE was detected in the ASG-10 and ASG-13 cells infected with the virus either on the 96-well plates or on coverslips in the 24-well plates.

**Table 1:** Virus susceptibility of five cell lines expressed as the calculated log_10_ TCID_50_ titer per 50 μl of a stock culture of six salmonid viruses.

	Virus					
Cell line	IHNV	VHSV	IPNV	TSRV	PSPV	ISAV
ASG-10	**≥7.3**	**≥7.0**	**6.3**	**5.5**	**4.0**	**0**
ASG-13	**≥7.0**	**≥7.3**	**5.7**	**6.5**	**3.7**	**0**
CHSE-214	**≥7.0**	**≥7.5**	**5.7**	**5.3**	**4.5**	**0**
ASK	**n.d**	**n.d**.	**n.d**.	**n.d**.	**n.d**.	**6.7**
EPC	**≥7.5**	**≥7.5**	**0**	**5.3**	**0**	**0**

#### Immunostaining for ISAV

The IFAT staining for ISAV NP on the coverslips revealed positive cells (Fig 1h and 1i) indicating that Glesvær/2/90, Can/F678/99 and FO/121/14 have the ability to infect a sub-population of the ASG-10 (Fig 1h) and ASG-13 cells (Fig 1i). More cells were positive in the wells infected with Can/F678/99 and FO/121/14 compared to the wells infected with Glesvær/2/90. Several of the infected cells appeared elevated compared to the uninfected cells. The uninfected cells appeared to be either pushing the infected cells away, or closing the hole or gap that would be made when the infected cells detached from the coverslip surface.

#### PCR for ISAV

All uninfected controls were negative. In ASG-10 infected with Glesvær/2/90 a reduction in Ct (cycle threshold) from 22.80 to 20.56 was seen from day 3 to day 7 after infection indicating a low level increase in viral load. In ASG-10 infected with Can/F678/99 a reduction in Ct from 25.86 to 22.16 was seen from day 0 to day 7. In ASG-10 infected with FO/121/14 a reduction in Ct from 22.11 to 20.69 was seen from day 3 to day 7. In ASG-13 infected with Glesvær/2/90 a reduction in Ct from 23.35 to 20.15 was seen from day 0 to day 7. No marked reduction in Ct was seen in ASG-13 infected with either Can/F678/99 or FO/121/14.

### Migratory and proliferative abilities

**Fig 2:**
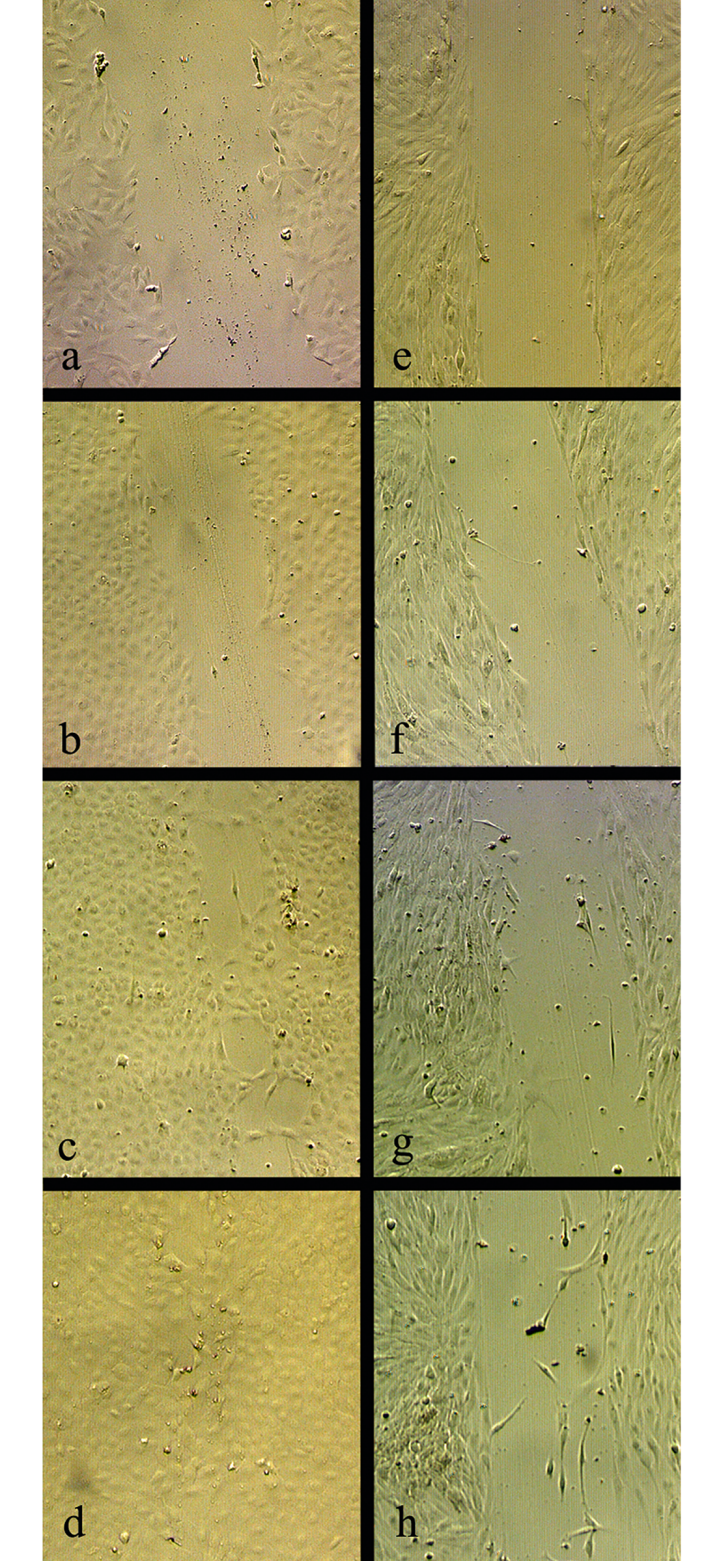
Cell culture migratory and proliferative properties. Pictures were taken daily during a 7 days scratch closure assay. Representative pictures of ASG-10 (a-d) and ASG-13 (e-h) at day 0 (a and e), day 3 (b and f), day 6 (c and g) and day 7 (d and h) are shown.

**Fig 3:**
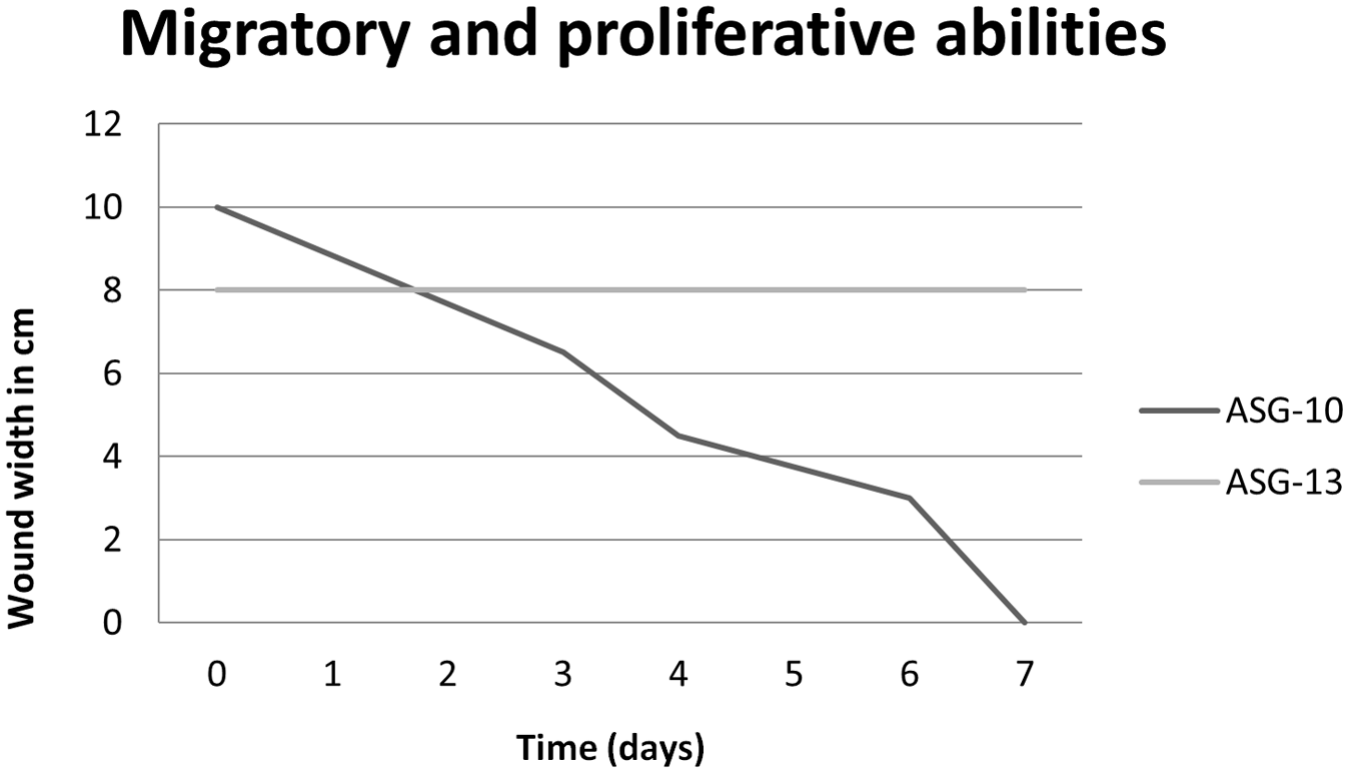
Measurement of scratch width. Relative measurement of the width of the scratch in cm to assess repair abilities of the two cell lines. The distance of the scratch was measured in cm on enhanced snapshots. The plot displays the width of the scratch over time.

During the seven days of the assay to investigate migratory and proliferative abilities of ASG-10 and ASG-13, pictures were taken daily. Representative pictures are shown in Fig 2. Relative measurement of width of the scratch was preformed to investigate the abilities of the two cell lines (Fig 3). ASG-10 migrated and closed the scratch gradually. At day 7 the scratch was completely closed. ASG-13 had not closed the scratch, and the width of the damaged monolayer was the same after 7 days, however some cells appeared to be migrating into the scratch from day 6.

## Discussion

Here we describe the establishment and characterization of two adherent cell lines from gill explants from Atlantic salmon. In one culture, the confluent cells express morphological surface structures consistent with epithelium. The other culture has a fibroblastic appearance. They have been maintained for 2.5 years through almost 30 passages and are confirmed to originate from Atlantic salmon. Both cell lines are fully susceptible to infection by IHNV, VHSV, IPNV, TSRV and PSPV as judged by appearance of typical CPE while sub-fractions of the cells appeared permissive for ISAV by immunofluorescence. The ASG-10 line is able to proliferate and migrate to close a scratch in a monolayer within 7 days.

### Demonstration of epithelial cell hallmarks

Although criteria for characterizing a piscine epithelial cell line have not been developed, as they have for mammalian epithelial cell lines [13], several papers describe cultures of gill epithelial cells from fish, including Atlantic salmon [12, 20–25]. Because neither the ASG-10 nor ASG-13 cell line is cloned, some variation in morphology is expected. The polygonal shape of ASG-10 generated in this study, is consistent with epithelium and is confirmed by the presence of both desmosomes as visualized by TEM and by two adherence junctional proteins, *i*.*e*. ZO-1 and e-cadherin, as visualized by immunostaining. However, at passage 11 a sub-population of the ASG-13 cells was positive for cytokeratin. This may indicate that a sub-population of the ASG-13 is of epithelial origin. Our results correspond with those of Butler and Nowak (2004) [12] who were able to establish epithelial and fibroblastic cells from Atlantic salmon gills. In their study, antibodies to mammalian cytokeratins were used to visualize the epithelial cells in culture, but presence of adherence junctional proteins, the hallmark of epithelial cells [26], was not reported.

### Different success from different explants, possible explanations

The explants used in this study were collected at a fixed time from the same fish, however, the outgrowth varied from explant to explant. Slightly different anatomical parts of the gills were included in the wells, probably with different proliferative capacities. Undifferentiated epithelial cells in the primary lamellae are thought to be the proliferating pool of cells that *in vivo* give rise to the epithelium in the secondary lamellae [27] and this population could be the source of cells that form the projections in the explants *in vitro*. The growth was most vigorous from the edge of the explants cut with the scalpel, as should be expected when comparing this process to wound healing. Butler and Nowak (2004) [12] harvested gill cells by disaggregating the cells from the explant, using enzymes, demonstrating that different approaches can be used to establish cell cultures from Atlantic salmon gills.

### Virus susceptibility

Virus susceptibility assays demonstrated that both ASG-10 and ASG-13 were permissive for selected salmonid viruses; however, the lack of typical CPE in the ASG-10 or ASG-13 cells infected with ISAV indicates that CPE alone should not be used as means for determining virus permissiveness. In the present study, ISAV isolates had the ability to infect a sub-population of the cultured cells, as shown by positive immunostaining; however, the FO/121/14 and Can/F679/99 isolates appeared to infect a higher number of cells than did the Glesvær/2/90 isolate in the ASG-10 cells. This is similar to results from fish in experimental infection trials [16, 17] in which the three isolates have the ability to infect epithelial cells in the gills; however, the Can/F679/99 and FO/121/14 isolates have a higher prevalence in the gill and infect a higher number of epithelial cells, appearing more epitheliotropic than does the Glesvær/2/90 isolate. A reduction in Ct-values in the media between two time-points is a clear indication of replication. This combined with the positive nucleoprotein labelling show that ASG-10 and 13 are permissive to ISAV. However, ASG-10 appears to better support replication of the isolates with lower virulence than the ASG-13.

That only a sub-population of the ASG-10 or ASG-13 cells became infected with ISAV may indicate that the cells are in different stages of development and that ISAV only has the ability to infect certain cell stages. A more plausible explanation is that the cell lines contain a mix of different cell lineages because the ASG-10 and ASG-13 lines have not been cloned or purified in another way. In general, primary cells and cell lines in the early stages of development may contain more than one cell type, because of the heterogeneous nature of animal organs. The use of these mixed cultures in studies may lead to problems when interpreting the results. Although the cell lines appear stable, they should be re-evaluated in future experiments. However, mixed cells may also be an advantage, because they mimic reality better than a pure cloned cell strain. By cloning a cell line several traits may be lost. If the goal is to cultivate an agent, cloning may be a disadvantage, however if the goal is to study the specific pathways in certain cells or a specific response to a drug the cells should be cloned or selected in another way first.

### Migratory and proliferative properties

The adherent cells appeared to be migrating towards scratches in the cell layer thus closing the scratch. Proliferation and migration are known properties of epithelial cells and migratory assay revealed a difference in migratory abilities between the cells supporting the morphological characterization of the cells. The ability of ASG-10 to successfully close the scratch within seven days supports their epithelial nature and could represent an epithelial-mesenchymal transition [28]. ASG-13 did not have the ability to close the scratch, however the few cells that migrated slowly into the damaged monolayer is in accordance with their fibroblastic nature. Fibroblasts are known to be able to migrate slowly as individual cells [29].

## Conclusions

We have established and characterized two new adherent cell lines from Atlantic salmon. Future use of these cells may include cultivating other epitheliotropic agents, including viruses. In addition, we may be able to use the cells to study regeneration in the gills as a result of disease or injury. In general, *in vitro* cell culture systems represent a major alternative to *in vivo* animal testing. New epithelial cell lines will provide a tool to study host responses without sacrifice of fish as an alternative to animal testing. In addition, these two cell lines with epithelial and mesenchymal properties respectively from the same individual fish could be useful for studies of basic cell biology.
